# Epidemiology and genotypes analysis of human papillomavirus infection in Beijing, China

**DOI:** 10.1186/s12985-024-02292-3

**Published:** 2024-01-16

**Authors:** Jiao Wang, Haotian Li, Jieqiong Zhang, Hui Wang, Ying Li, Zhaohui Liu, Hongtu Liu

**Affiliations:** 1 National Institute for Viral Disease Control and Prevention, Chinese Center for Disease Control and Prevention, Beijing, 102206 China; 2 Department of Gynecology, Beijing Obstetrics and Gynecology Hospital, Beijing Maternal and Child Health Care Hospital, Capital Medical University, Beijing, 100026 China

**Keywords:** Cervical cancer, High-risk HPV, Cervical lesions, Ages, Cluster analysis

## Abstract

**Background:**

This study aimed to investigate the epidemiology of high-risk human papillomavirus (HPV) in the female population in Beijing, China, and identify the relationship between HPV genotypes and host factors.

**Methods:**

HPV testing was performed on women aged 15–89 (mean age 38.0 ± 10.9 years) from Beijing in 2020. High-risk HPV genotyping real-time polymerase chain reaction was used to determine HPV genotypes. The overall prevalence, age-specific prevalence, genotype distribution, and the correlation between HPV genotypes and cervical cytology were analyzed.

**Results:**

Among the 25,344 study participants, the single and double infection rates were 18.8% (4,777/25,344) and 4.2% (1,072/25,344), respectively. A total of 6,119 HPV-positive individuals were found to have 91.6% negative results for intraepithelial lesion or malignancy (NILM), 5.8% atypical squamous cells of undetermined significance (ASC-US), 0.9% low-grade squamous intraepithelial lesion (LSIL), and 1.7% high-grade squamous intraepithelial lesion (HSIL). In single HPV infections, the HPV16 genotype was highly associated with cervical cytology severity (χ^2^ trend = 172.487, *P* < 0.001). Additionally, HPV infection rates increased gradually with age, and statistical differences were observed across age groups (χ^2^ = 180.575; *P* < 0.001). High-risk HPV genotypes were highly prevalent in women below 25 years of age and those aged 55–59 years. Cluster analysis revealed that the 13 HPV genotypes could be roughly divided into two groups in a single infection; however, patterns of infection consistent with biological characteristics were not observed.

**Conclusion:**

High-risk HPV was found in 24.1% of outpatients, with HPV52, HPV58, HPV16, HPV39, and HPV51 being the most common high-risk genotypes. Single high-risk HPV infection was predominant. HPV16, HPV39, HPV51, and HPV52 were associated with cervical lesion progression. HPV16 infection was especially worrying since it aggravates cervical lesions. Because the infection rates of the 13 HPV genotypes differed by age, the peak HPV infection rate should not guide vaccination, screening, and prevention programs. Instead, these initiatives should be tailored based on the regional HPV distribution characteristics. Moreover, it was determined that Beijing’s populace needed to receive treatment for HPV39 infection.

## Introduction

Cervical cancer is one of the most common cancers in women worldwide. In 2020, there were about 342,000 deaths and about 604,000 new cases identified worldwide, with a large share of these deaths taking place in low- and middle-income nations [1]. The persistence of human papillomavirus (HPV) infection is widely recognized as the most important causative factor in the development of cervical cancer [2].

The genome of HPV, a small, double-stranded circular DNA virus, is about 8 kb [3]. Over 200 HPV genotypes have been identified based on genomic differences, and most genotypes are harmless [4]. There is clear evidence linking the human papillomavirus (HPV) to cervical cancer: the incidence of cervical cancer is highly correlated with the frequency of high-risk HPV [5]. Thirteen HPV genotypes (HPV16, 18, 31, 33, 35, 39, 45, 51, 52, 56, 58, 59, and 68) that are designated high-risk HPV are essential factors for cervical tumorigenesis [6]. The five most common HPV types in women worldwide who are HPV-positive are HPV16, 18, 31, 58, and 52. However, the ranking of the prevalence of these types varies by region [7]. For instance, in Western Europe, HPV16, 18, 31, 35, and 33, and in North America, HPV16, 53, 18, 51, and 31 are the five most prevalent high-risk HPV strains [8]. HPV16, 52, and 58 have the highest infection rates in some areas of China [9–12].

The predominant HPV genotypes may cause different outcomes at different stages of the disease [13]. Negative for intraepithelial lesion or malignancy (NILM), atypical squamous cells of undetermined significance (ASC-US), low-grade squamous intraepithelial lesion (LSIL) and high-grade squamous intraepithelial lesion (HSIL) are the four categories used to categorize precancerous phases [14]. Among cases of HSIL, the prevalence of HPV16/18 is 52%. However, HPV 16, 18, and 45 are significantly under-represented, and other high-risk HPV types are significantly over-represented in HSIL compared with invasive cervical cancer, suggesting differences in type-specific risks for progression [15]. The most common HPV types in HSIL samples from China are HPV16, 58, 52, 18, and 33 [16].

Furthermore, age has a significant role in determining the risk of HPV infection [17]. In Western countries, HPV prevalence peaks only in women in their mid-twenties and then steadily declines as age increases [18]. However, the prevalence of high-risk HPV has two peaks in China: one at age 15–24 years and the other at age 35–49 years [19].

To investigate the state of high-risk HPV infection, the correlation between high-risk HPV genotypes and cervical lesion severity, and the genotype distribution in the area, 25,344 samples from female outpatients in Beijing, China, were gathered for this study in 2020. We aimed to investigate cervical cancer epidemiology, diagnosis, and vaccination in Beijing, China.

## Materials and methods

### Study population

The study’s data came from patients who visited Beijing Obstetrics and Gynecology Hospital, Capital Medical University’s gynecological outpatient department in 2020. After the exclusion of unqualified samples, the quantitative measurement and biopsy of high-risk HPV DNA from a total of 25,344 women was performed. The median age was 38.0 ± 10.9 years, and the age range was 15–89 years (Table 1). The study was approved by the ethics committee of Beijing Obstetrics and Gynecology Hospital, Capital Medical University, and written informed consent was acquired from the study participants.

### Cervical specimen collection and high-risk HPV genotyping

Following instructions, women’s cervical exfoliated cell samples were obtained using cytobrushes, and the samples were utilized to extract genomic DNA. DNA was isolated using a nucleic acid extraction reagent (Shanghai ZJ Bio-Tech Co., Ltd., Shanghai, China). Then, a commercial HPV genotyping kit (Shanghai ZJ Bio-Tech Co., Ltd.) was used to detect 13 high-risk HPV types (HPV16, 18, 31, 33, 35, 39, 45, 51, 52, 56, 58, 59, and 68) with use of TaqMan real-time fluorescent quantitative polymerase chain reaction. The kit’s instructions were strictly followed for every procedure.

### ThinPrep cytologic test

Cervical cells were detected using the ThinPrep cytology test (TCT). Senior physicians assessed cytological pathology results according to the Bethesda System of Cervical Cytology, which classifies precancerous phases as NILM, ASC-US, LSIL, or HSIL. Histopathological diagnoses were made by a pathologist who was unaware of the HPV detection results.

### Cluster analysis

We investigated the similarity of infection for 13 high-risk HPV types in abnormal cytology across age groups using cluster analysis. We considered that the classical K-means algorithm was suitable for this study. K-means used Manhattan distance to measure the distance between two observations. Hierarchical cluster analysis was conducted using a “hclust” function in R-Studio (Version 4.2.1).

### Statistical analysis

All statistical analyses performed in this study were with R software version 4.2.1. Figures were created with GraphPad Prism version 9 (GraphPad Software, San Diego, CA, USA). A binomial 95% confidence interval (CI) was calculated for each calculation used to estimate the prevalence of HPV, with separate computations made for each genotype from single and multiple infections. These data were further stratified by age (< 20, 20–24, 25–29, 30–34, 35–39, 40–44, 45–49, 50–54, 55–59, and ≥ 60 years). Differences between groups were tested using the Pearson χ^2^ test depending on data type and distribution. Differences were considered statistically significant if *P* < 0.05.

## Results

### Prevalence of high-risk HPV genotypes in the cervical samples

The high-risk HPV infection rate among the 25,344 patients was 24.1% (6,119/25,344), with 6,119 participants showing positive findings from high-risk HPV tests. Among the women who were HPV-positive, 4,777 were positive for a single HPV type (4,777/6,119 = 78.1%, 4,777/25,344 = 18.8%). Furthermore, 1,342 were positive for multiple types (1,342/6,119 = 21.9%, 1,342/25,344 = 5.3%), of which 1,072 were positive for two types (1,072/6,119 = 17.5%, 1,072/25,344 = 4.2%; Table 1). Among single HPV infections, the five most prevalent high-risk types were HPV52, 58, 16, 39, and 51, with prevalences of 3.7%, 3.0%, 2.8%, 1.8%, and 1.7%, respectively. HPV18 had a prevalence of 1.0% (246/25,344) and was ranked seventh. Among 1,342 individuals infected with multiple HPV types, the five most prevalent high-risk HPV types were HPV52, 39, 58, 16, and 51, with prevalences of 1.7%, 1.6%, 1.6%, 1.5%, and 1.0%, respectively. In addition, HPV68 ranked seventh in prevalence among multiple infections (0.8%; Table 2). The five high-risk types with the highest overall infection rates were HPV52 (1,385/25,344, 5.5%, 95% CI 5.2–5.7), 58 (1,161/25,344, 4.6%, 95% CI 4.3–4.8), 16 (1,099/25,344, 4.3%, 95% CI 4.1–4.6), 39 (865/25,344, 2.7%, 95% CI 3.2–3.6), and 51 (697/25,344 2.8%, 95% CI 2.6-3.0). Moreover, the five high-risk types with the lowest overall infection rates were HPV31 (280/25,344 1.1%, 95% CI 1.0-1.2), 68 (267/25,344, 1.1%, 95% CI 0.9–1.2), 33 (252/25,344, 1.0%, 95% CI 0.9–1.1), 35 (244/25,344, 1.0%, 95% CI 0.8–1.1), and 45 (124/25,344, 0.5%, 95% CI 0.4–0.6; Table 2).

**Table 1:** Information about women with high-risk human papillomavirus infection

High-risk HPV-positive women (%) *n* = 25,344		95% Cl
Mean ± SD	38.0 ± 10.9	37.9–38.2
Age range	15–89	
**Infection type (no. of women)**		
All	6,119 (24.1)	23.6–24.7
Single	4,777 (18.8)	18.4–19.3
Multiple	1,342 (5.3)	5.0–5.6
Double	1,072 (4.2)	4.0–4.5

**Table 2:** Distribution of HPV genotypes in 25,344 women with high-risk human papillomavirus infection

HPV	Case no.(*n* = 25,344)		Single infection				Multiple infections			Double infection		
type	Positive no. (%)	95% Cl	Positive no. (%)		95% Cl		Positive no. (%)		95% Cl	Positive no. (%)	95% Cl	
16	1,099 (4.3)	4.1–4.6	721 (2.8)		2.6–3.1		378 (1.5)		1.3–1.6	277 (1.1)	1.0–1.2	
18	417 (1.7)	1.5–1.8	246 (1.0)		0.9–1.1		171 (0.7)		0.6–0.8	122 (0.5)	0.4–0.6	
31	280 (1.1)	1.0–1.2	177 (0.7)		0.6–0.8		103 (0.4)		0.3–0.5	68 (0.3)	0.2–0.3	
33	252 (1.0)	0.9–1.1	154 (0.6)		0.5–0.7		98 (0.4)		0.3–0.5	65 (0.3)	0.2–0.3	
35	244 (1.0)	0.8–1.1	134 (0.5)		0.4–0.6		110 (0.4)		0.6 − 0.5	77 (0.3)	0.2–0.4	
39	865 (3.4)	3.2–3.6	463 (1.8)		1.7–2.0		402 (1.6)		1.4–1.7	297 (1.2)	1.0–1.3	
45	124 (0.5)	0.4–0.6	79 (0.3)		0.2–0.4		45 (0.2)		0.1–0.2	28 (0.1)	0.1–0.2	
51	697 (2.8)	2.6–3.0	436 (1.7)		1.6–1.9		261 (1.0)		0.9–1.2	190 (0.8)	0.6–0.9	
52	1,385 (5.5)	5.2–5.7	943 (3.7)		3.5–4.0		442 (1.7)		1.6–1.9	315 (1.2)	1.1–1.4	
56	602 (2.4)	2.2–2.6	354 (1.4)		1.3–1.5		248 (1.0)		0.9–1.1	171 (0.7)	0.6–0.8	
58	1,161 (4.6)	4.3–4.8	769 (3.0)		2.8–3.3		392 (1.6)		1.4–1.7	278 (1.1)	1.0–1.2	
59	390 (1.5)	1.4–1.7	226 (0.9)		0.8–1.0		164 (0.7)		0.6–0.8	108 (0.4)	0.4–0.5	
68	267 (1.1)	0.9–1.2	75 (0.3)		0.2–0.4		192 (0.8)		0.7–0.9	148 (0.6)	0.5–0.7	

### Cervical cytological status of age groups and high‑risk genotypes

The prevalence of HPV in women with ASC-US was found to be 45.5% (357/785) in the cytologically abnormal samples, but in women with LSIL and HSIL, the rates were 78.3% (54/69) and 78.9% (105/133), respectively. The cervical cytological status of the 6,119 HPV-positive individuals was analyzed: 91.6% had NILM, 5.8% had ASC-US, 0.9% had LSIL, and 1.7% had HSIL, respectively (Table 3). The data were then divided by the following age groups: < 20, 20–24, 25–29, 30–34, 35–39, 40–44, 45–49, 50–54, 55–59, and ≥ 60 years. Among women with NILM, the 25-29-year age group had the highest rate (96.1%), whereas the < 20-year age group had the lowest rate (82.4%). The < 20-year age group (17.6%) had the highest rate among women with ASC-US. In women with LSIL and HSIL, the 35-39-year group and the ≥ 60-year age group had a high prevalence. The findings suggest that those who are HPV-positive may have a progressive increase in their risk of cervical carcinogenesis beyond the age of 35.

**Table 3:** Distribution of cervical cytological status by age group

Age group	Positive no.	NILM (*n* = 24,357) Positive no. (%)	ASC-US (*n* = 785) Positive no. (%)	LSIL (*n* = 69) Positive no. (%)	HSIL (*n* = 133) Positive no. (%)
< 20	34	28(82.4)	6(17.6)	0(0)	0(0)
20–24	357	339(95.0)	16(4.5)	1(0.3)	1(0.3)
25–29	1,241	1,192(96.1)	40(3.2)	7(0.6)	2(0.2)
30–34	1,279	1,191(93.1)	62(4.8)	10(0.8)	16(1.3)
35–39	818	748(91.4)	43(5.3)	8(1.0)	19(2.3)
40–44	748	653(87.3)	67(9.0)	9(1.2)	19(2.5)
45–49	594	518(87.2)	50(8.4)	7(1.2)	19(3.2)
50–54	475	430(90.5)	34(7.2)	4(0.8)	7(1.5)
55–59	334	302(90.4)	19(5.7)	4(1.2)	9(2.7)
≥ 60	239	202(84.5)	20(8.4)	4(1.7)	13(5.4)
Total	6,119	5,603(91.6)	357(5.8)	54(0.9)	105(1.7)

### The relationship between high-risk HPV genotypes and abnormal cervical cytology

We examined the connection between TCT outcomes and high-risk HPV genotypes in HPV-positive people. The results showed that the association between single and multiple infections and abnormal cytology differed for each genotype. Among single infections, the top five genotypes among women with NILM were HPV52 (20.3%), 58 (16.5%), 16 (13.5%), 39 (10.0%), and 51 (9.4%). The most prevalent HPV types in HSIL were as follows: HPV16 (63.0%), 58 (7.4%), 33 (6.2%), 31 (4.9%), 52 (4.9%), and 18 (3.7%; Table 4). The overall single infection rate increased with cervical cytological status. The infection rate of HPV16 showed a gradual increase with disease progression, whereas the infection rates of HPV31, 33, and 45 were slightly higher in HSIL than in NILM samples. The infection rates of HPV18, 35, 39, 51, 52, 56, 58, 59, and 68 showed a decreasing trend. In the case of abnormal TCT, the infection rate of HPV16 (χ^2^ trend = 172.487, *P* < 0.001) increased with increasing cervical cytological severity, whereas the infection rates of HPV39 (χ^2^ trend = 8.569, *P* = 0.003), 51 (χ^2^ trend = 7.708, *P* = 0.005), and 52 (χ^2^ trend = 16.949, *P* < 0.001) showed the opposite trend (Table 4). An examination of the cervical cytological state and HPV genotype was not conducted due to the lack of data on multiple infections.

**Fig. 1:**
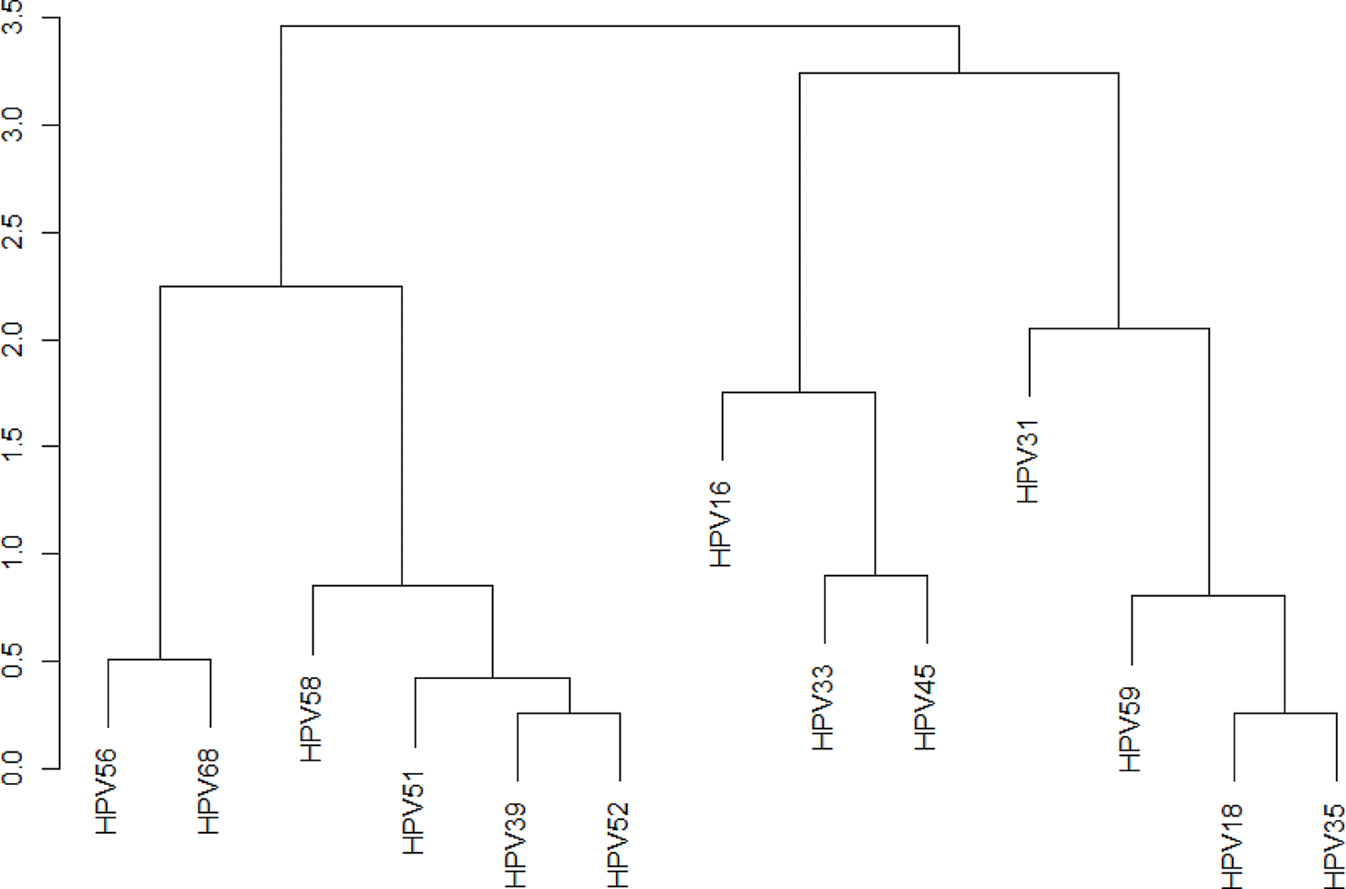
Cluster analysis of high-risk HPV genotypes and cervical cytological status

Two categories of HPV genotypes may be identified by cluster analysis based on the trend of high-risk HPV genotypes by cervical cytological status [20]. HPV16, 18, 31, 33, 35, 45, and 59 had similar infection trends, whereas HPV39, 51, 52, 56, 58, and 68 had similar infection trends (Fig. 1). The result is associated with the risk estimates of high-risk HPV genotypes in tumors.

**Table 4:** The relationship between high-risk genotypes and TCT results in single HPV-positive specimens

HPV type	NILM (*n* = 4,422) Positive no. (%)	ASC-US (*n* = 248) Positive no. (%)	LSIL (*n* = 33) Positive no. (%)	HSIL (*n* = 80) Positive no. (%)	χ2 (P)	χ2_trend_ (P)
16	597 (13.5)	63 (25.4)	10 (30.3)	51 (63.0)	**179.871 (0.001)**	**172.487 (0.001)**
18	228 (5.2)	14 (5.6)	1 (3.0)	3 (3.7)	0.777 (0.855)	0.266 (0.606)
31	157 (3.6)	15 (6.0)	1 (3.0)	4 (4.9)	4.479 (0.214)	1.877 (0.171)
33	136 (3.1)	13 (5.2)	0 (0.0)	5 (6.2)	6.886 (0.076)	3.096 (0.078)
35	123 (2.8)	10 (4.0)	0 (0.0)	1 (1.2)	3.061 (0.382)	0.242 (0.623)
39	443 (10.0)	16 (6.5)	2 (6.1)	2 (2.5)	**8.890 (0.031)**	**8.569 (0.003)**
45	74 (1.7)	3 (1.2)	0 (0.0)	2 (2.5)	1.200 (0.753)	0.003 (0.959)
51	415 (9.4)	18 (7.3)	2 (6.1)	1 (1.2)	**7.898 (0.048)**	**7.708 (0.005)**
52	898 (20.3)	37 (14.9)	4 (12.1)	4 (4.9)	**17.050 (0.001)**	**16.949 (0.001)**
56	330 (7.5)	15 (6.0)	7 (21.2)	2 (2.5)	12.741 (0.005)	0.550 (0.458)
58	727 (16.5)	31 (12.5)	5 (15.2)	6 (7.4)	7.372 (0.061)	6.667 (0.010)
59	215 (4.9)	11 (4.4)	0 (0.0)	0 (0.0)	5.898 (0.117)	5.007 (0.025)
68	72 (1.6)	2 (0.8)	1 (3.0)	0 (0.0)	2.789 (0.425)	1.395 (0.238)

### Age-specific prevalence of HPV infection

The participants were divided into ten groups by age category: <20, 20–24, 25–29, 30–34, 35–39, 40–44, 45–49, 50–54, 55–59, and ≥ 60 years. The prevalence of high-risk HPV infection was significantly different across age groups (χ^2^ = 180.575; *P* < 0.001). In this study, the “two peaks” pattern was observed for the prevalence of HPV infection: the prevalence of overall HPV genotypes showed a first peak at age < 20 years (55.7%, 34/61) and a second peak at age 55–59 years (30.1%, 334/1,108; Table 5). We compiled the infection status of 13 high-risk HPV genotypes across 10 age groups in this investigation (Table 6). The age of the second peak varied among high-risk HPV genotypes; for HPV16 and HPV18, the second infection peak occurred after age 35 years, and for HPV31 and HPV51, it occurred at age 50–54 years. Despite this, the prevalence of all HPV genotypes increased once more to form a second peak at age 55–59 years.

**Fig. 2:**
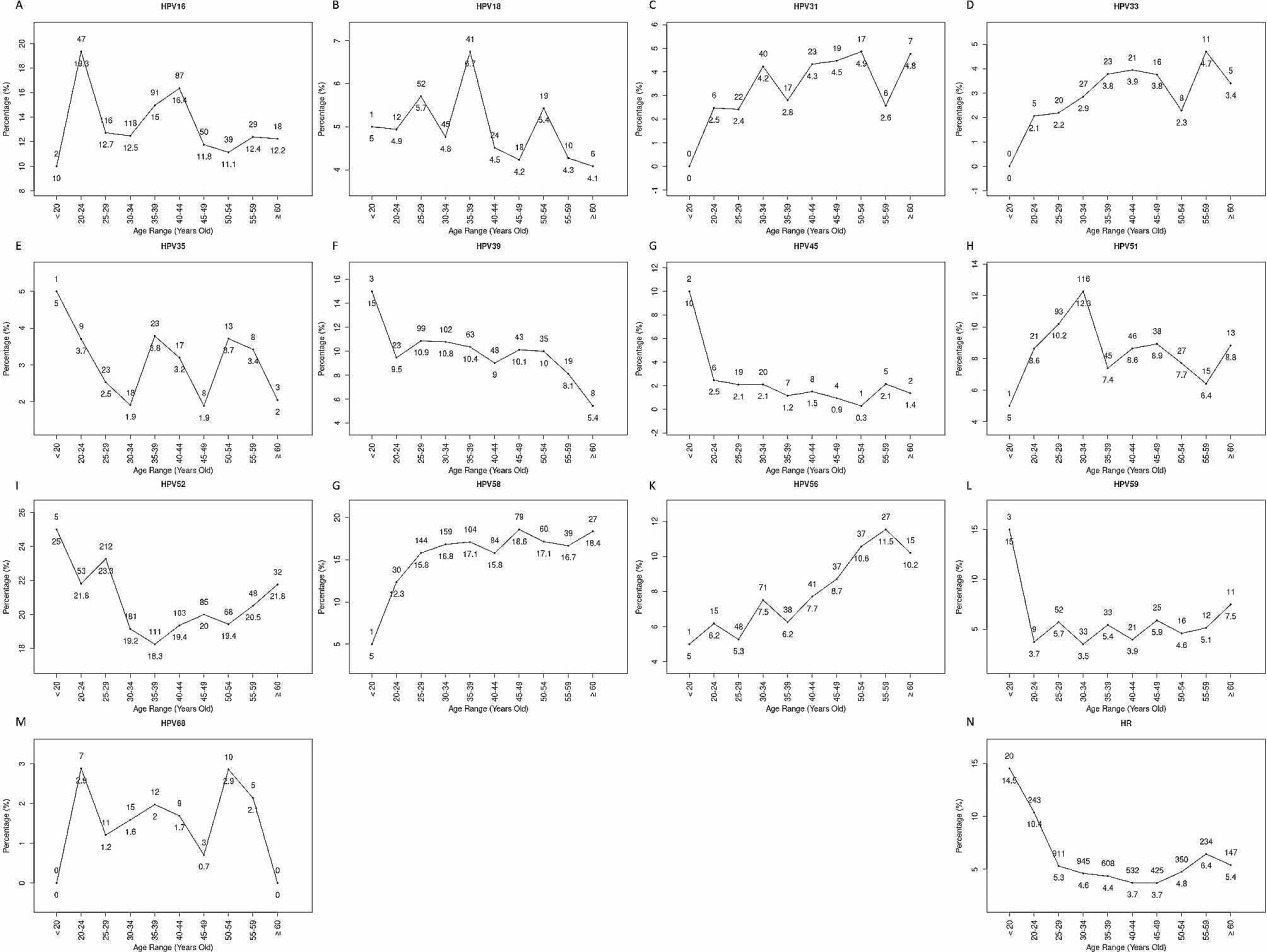
The prevalence by age of each high-risk HPV genotype in a single HPV infection

In addition, we observed differing trends for HPV genotypes and age groups for single and multiple infections. We concentrated on the distribution of each HPV genotype in single infections solely because numerous factors affect multiple infections. Among the single infections, the first peak was observed in young women (15–25 years old), whereas the second peak was observed at different ages. HPV31, 33, 45, 51, 52, 56, and 68 had two infection peaks, and HPV16, 18, 35, 39, 58, and 59 had three or more infection peaks. Before the ages of 45 and 40, respectively, the infection rates for HPV33 and 58 rose with age. HPV16, 33, 45, and 56 had re-emerge peaks at 55–59 years of age; HPV18, 31, 35, 39, and 68 at 50–54 years of age; HPV51, 58, and 59 at 45–49 years of age; and HPV52 infection increased with age from 35 years of age (Fig. 2).

**Fig. 3:**
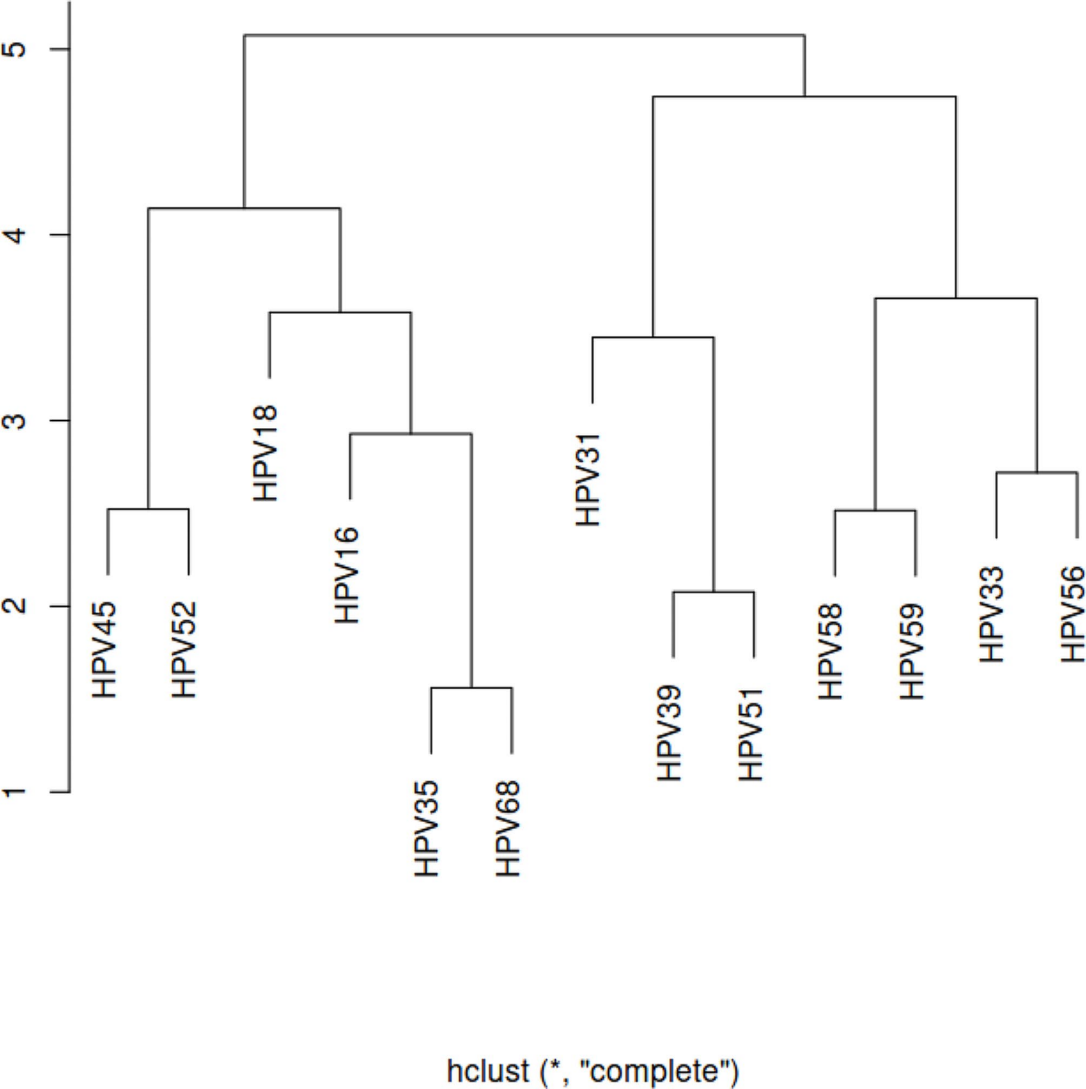
Cluster analysis of HPV genotypes in single infection across age groups

HPV genotypes may be separated into two groups depending on the age group infection rate, according to cluster analysis based on the trend of the percentage change of HPV genotypes among patients who were HPV-positive in each age group. HPV16, 18, 35, 45, 52, and 68 had similar infection trends, whereas HPV31, 33, 39, 51, 56, 58, and 59 had similar infection trends (Fig. 3). However, the infection trend of HPV genotypes in age groups was not significantly associated with biological characteristics. The correlates of infection trends were unclear.

**Table 5:** Distribution of HPV infection by age group in 25,344 women with high-risk HPV infection

Age group	Cases no.	Positive no.		HPV Positive Prevalence (%)	95% Cl	χ^2^ (P)
		Single infection	Multiple infections			
< 20	61	23	11	34 (55.7)	42.9–68.6	180.575 (< 0.001)
20–24	950	252	105	357 (37.6)	34.5–40.7	
25–29	5,383	938	303	1,241 (23.1)	21.9–24.2	
30–34	5,499	1,010	269	1,279 (23.3)	22.1–24.4	
35–39	3,361	661	157	818 (24.3)	22.9–25.8	
40–44	3,455	608	140	748 (21.6)	20.3–23.0	
45–49	2,666	479	115	594 (22.3)	20.7–23.9	
50–54	1,776	384	91	475 (26.7)	24.7–28.8	
55–59	1,108	254	80	334 (30.1)	27.4–32.9	
≥ 60	1,085	168	71	239 (22.0)	19.6–24.5	

**Table 6:** Age distribution of 13 high-risk HPV types

HPV type	< 20 (*n* = 61)		20–24 (*n* = 950)		25–29 (*n* = 5,383)		30–34 (*n* = 5,499)		35–39 (*n* = 3,361)		40–44 (*n* = 3,455)		45–49 (*n* = 2,666)		50–54 (*n* = 1,776)		55–59 (*n* = 1,108)		≥ 60 (*n* = 1,085)	
	n. (%)	95% CI	n. (%)	95% CI	n. (%)	95% CI	n. (%)	95% CI	n. (%)	95% CI	n. (%)	95% CI	n. (%)	95% CI	n. (%)	95% CI	n. (%)	95% CI	n. (%)	95% CI
16	5 (8.2)	1.3–15.1	79 (8.3)	6.6–10.1	210 (3.9)	3.4–4.4	212 (3.9)	3.3–4.4	174 (5.2)	4.4–5.9	152 (4.4)	3.7–5.1	100 (3.8)	3–4.5	68 (3.8)	2.9–4.7	55 (5.0)	3.7–6.2	44 (4.1)	2.9–5.2
18	4 (6.6)	0.3–12.8	25 (2.6)	1.6–3.6	109 (2.0)	1.6–2.4	81 (1.5)	1.2–1.8	61 (1.8)	1.4–2.3	42 (1.2)	0.9–1.6	32 (1.2)	0.8–1.6	31 (1.7)	1.1–2.4	15 (1.4)	0.7–2.0	17 (1.6)	0.8–2.3
31	1 (1.6)	-1.5–4.8	10 (1.1)	0.4–1.7	46 (0.9)	0.6–1.1	56 (1.0)	0.8–1.3	34 (1.0)	0.7–1.3	40 (1.2)	0.8–1.5	35 (1.3)	0.9–1.7	30 (1.7)	1.1–2.3	11 (1.0)	0.4–1.6	17 (1.6)	0.8–2.3
33	3 (4.9)	-0.5–10.3	20 (2.1)	1.2–3.0	32 (0.6)	0.4–0.8	52 (0.9)	0.7–1.2	41 (1.2)	0.8–1.6	38 (1.1)	0.8–1.4	21 (0.8)	0.5–1.1	11 (0.6)	0.3–1.0	19 (1.7)	1.0–2.5	15 (1.4)	0.7–2.1
35	1 (1.6)	-1.5–4.8	16 (1.7)	0.9–2.5	51 (0.9)	0.7–1.2	36 (0.7)	0.4–0.9	40 (1.2)	0.8–1.6	27 (0.8)	0.5–1.1	20 (0.8)	0.4–1.1	23 (1.3)	0.8–1.8	19 (1.7)	1.0–2.5	11 (1.0)	0.4–1.6
39	8 (13.1)	4.6–21.6	56 (5.9)	4.4–7.4	196 (3.6)	3.1–4.1	177 (3.2)	2.8–3.7	109 (3.2)	2.6–3.8	101 (2.9)	2.4–3.5	92 (3.5)	2.8–4.1	58 (3.3)	2.4–4.1	46 (4.2)	3.0–5.3	22 (2.0)	1.2–2.9
45	2 (3.3)	-1.2–7.7	11 (1.2)	0.5–1.8	33 (0.6)	0.4–0.8	28 (0.5)	0.3–0.7	11 (0.3)	0.1–0.5	13 (0.4)	0.2–0.6	8 (0.3)	0.1–0.5	6 (0.3)	0.1–0.6	10 (0.9)	0.3–1.5	2 (0.2)	-0.1–0.4
51	4 (6.6)	0.3–12.8	48 (5.1)	3.7–6.4	165 (3.1)	2.6–3.5	177 (3.2)	2.8–3.7	74 (2.2)	1.7–2.7	77 (2.2)	1.7–2.7	53 (2.0)	1.5–2.5	46 (2.6)	1.9–3.3	27 (2.4)	1.5–3.3	26 (2.4)	1.5–3.3
52	8 (13.1)	4.6–21.6	80 (8.4)	6.7–10.2	315 (5.9)	5.2–6.5	287 (5.2)	4.6–5.8	173 (5.1)	4.4–5.9	157 (4.5)	3.8–5.2	125 (4.7)	3.9–5.5	96 (5.4)	4.4–6.5	81 (7.3)	5.8–8.8	63 (5.8)	4.4–7.2
56	4 (6.6)	0.3–12.8	38 (4.0)	2.8–5.2	94 (1.7)	1.4–2.1	123 (2.2)	1.8–2.6	60 (1.8)	1.3–2.2	69 (2.0)	1.5–2.5	59 (2.2)	1.7–2.8	68 (3.8)	2.9–4.7	51 (4.6)	3.4–5.8	36 (3.3)	2.3–4.4
58	5 (8.2)	1.3–15.1	65 (6.8)	5.2–8.4	228 (4.2)	3.7–4.8	250 (4.5)	4.0–5.1	145 (4.3)	3.6–5.0	136 (3.9)	3.3–4.6	117 (4.4)	3.6–5.2	97 (5.5)	4.4–6.5	63 (5.7)	4.3–7.0	55 (5.1)	3.8–6.4
59	1 (1.6)	3.5–19.5	27 (2.8)	1.8–3.9	88 (1.6)	1.3–2.0	77 (1.4)	1.1–1.7	49 (1.5)	1.1–1.9	35 (1.0)	0.7–1.3	41 (1.5)	1.1–2	28 (1.6)	1.0–2.2	19 (1.7)	1.0–2.5	19 (1.8)	1.0–2.5
68	0 (0)	-1.5–4.8	21 (2.2)	1.3–3.1	60 (1.1)	0.8–1.4	53 (1.0)	0.7–1.2	33 (1.0)	0.6–1.3	27 (0.8)	0.5–1.1	29 (1.1)	0.7–1.5	20 (1.1)	0.6–1.6	18 (1.6)	0.9–2.4	5 (0.5)	0.1–0.9

## Discussion

Cervical cancer is one of the most preventable cancers. The primary cause of HSIL in the cervix and cervical cancer is high-risk HPV persistent infection. In 2018, the World Health Organization called for global action to eliminate cervical cancer and proposed that 90% of girls aged 9–14 years receive the HPV vaccine [21]. In most nations, a complete strategy that includes HPV vaccination and HPV-based screening is economical [22]. However, regional disparities in cervical cancer screening have been caused by the varying strengths of local governments and differing patient participation rates. China launched a national public health program to curb cervical cancer in 2009 [23]. However, immunization and cervical cancer screening rates are still low [24].

The frequency of high-risk HPV genotypes among Beijing Obstetrics and Gynecology Hospital outpatients was 24.1% in this study, which is in line with earlier studies [25, 26]. The results supported that HPV52, 58, and 16 are the most prevalent HPV genotypes among women in Beijing. Previous studies have demonstrated that HPV52 and 58 are the two dominant HPV genotypes in East Asia and China [27]. This study’s high-risk HPV-type prevalence is consistent with earlier studies [9, 28, 29]. A single genotype typically causes high-risk HPV infection; however, multiple infections have gradually gained attention in recent years. Infection with multiple HPV types is reported in 20–45% of women worldwide who are infected with HPV [30, 31]. Regarding the distinction between the effects of a single high-risk HPV infection and numerous high-risk infections on cervical precancer and cancer, there is no conclusive evidence [9, 32, 33]. In this study, high-risk HPV infection was mainly caused by a single genotype (18.8%), whereas multiple infections accounted for 5.3% of cases. Furthermore, we estimated that 1.4% (355/25,344) of outpatients were infected with low-risk HPV types (HPV6 and 11).

Recent studies indicate that HSIL, a significant stage of cervical precancerous lesions, and cervical cancer may also be caused by other high-risk HPV strains. This study provides an in-depth analysis of the relationship between cervical cytology and HPV infection. We found that the relationship between cervical cytological state and HPV type varied depending on whether the infection was single or multiple. In single infection, HPV16 was the most common type among women with HSIL, followed by HPV58, 33, 31, 52, and 18. Furthermore, the prevalence of HPV16, 39, 51, and 52 infections was strongly correlated with the severity of cervical cytology. The proportion of HPV16 gradually increased with disease progression. On the other hand, as the illness advanced, the proportions of HPV39, 51, and 52 steadily decreased. These results were consistent with previous findings [10, 34]. In multiple infections, high-risk HPV was less likely to progress to HSIL than in single infections. This study observed no effect of multiple infections on abnormal cervical cytology.

In addition, the prevalence of overall high-risk HPV infections displayed a bimodal age distribution, with one peak at ≤ 25 years, a decline with age, and a second peak at 55–59 years of age. However, the infection rates of different HPV genotypes differed across age groups. For instance, HPV-16 and 18 peaked again between the ages of 35 and 39. Consequently, we suggest that women over 35 be mandated to undergo an annual HPV test. Despite similar infection trends for single and multiple infections, we only analyzed the shifts in infection curves for single HPV infections because the etiologies of multiple infections are more complex. We found that the peak age of each HPV strain varied.

Furthermore, the 13 high-risk HPV types classified as oncogenic based on epidemiologic and/or phylogenetic evidence are members of four species within the Alpha-papillomavirus genus. HPV 16, 31, 33, 35, 52, and 58 are the prototypes of the A9 species; HPV 18, 39, 45, 68, and 59 are the prototypes of the A7 species; HPV51 is the prototype of the A5 species; and HPV56 is the prototype of the A6 species. Our study categorized the 13 HPV types by cluster analysis and finally divided them into two groups: (1) HPV16, 18, 35, 45, 52, and 68, which had a similar age distribution, and (2) HPV31, 33, 39, 51, 56, 58, and 59, which had similar infection trends. Nevertheless, no noteworthy correlation was detected with epidemiology and systems biology classification techniques.

Ultimately, immunization is one of the most important strategies for lowering cervical cancer incidence. Vaccines should be effectively and rationally distributed by region according to HPV epidemiological characteristics. Cervical cancer vaccination is considered an important measure for the effective prevention of cervical precancer, cervical cancer, and acromegaly. Moreover, variations in HPV subtype infection are noted between regions and ethnic groups. Therefore, a thorough big sample survey may offer useful therapeutic value for vaccine development and vaccination to prevent cervical cancer in pertinent places. The sample in this study reflects the high-risk HPV infection status of the Beijing population. The imported nine-valent HPV vaccine has prevented the HPV subtypes, including HPV6, 11, 16, 18, 31, 33, 45, 52, and 58 in the Beijing population. Notably, however, HPV39 was not covered by the vaccine. As a result, the vaccine’s ability to prevent high-risk HPV in Beijing is still limited.

## Conclusion

In conclusion, this study examined the frequency of high-risk HPV infection in females, the correlation between high-risk HPV genotypes and cervical lesion severity, and the association between high-risk HPV infection and age distribution features in Beijing, China in 2020. Our study will provide helpful information for screening and vaccinating cervical cancer in Beijing, China.

## Data Availability

The data was collected from Beijing Obstetrics and Gynecology Hospital, Capital Medical University. We thank them for their generous help.

## Abbreviations

HPV: Human papillomavirus
NILM: Negative for intraepithelial lesion or malignancy
ASC-US: Atypical squamous cells of undetermined significance
LISL: Low-grade squamous intraepithelial lesion
HSIL: High-grade squamous intraepithelial lesion
TCT: ThinPrep cytology test
